# Palladium Nanoparticles Induce Disturbances in Cell Cycle Entry and Progression of Peripheral Blood Mononuclear Cells: Paramount Role of Ions

**DOI:** 10.1155/2014/295092

**Published:** 2014-07-03

**Authors:** Claudia Petrarca, Emanuela Clemente, Luca Di Giampaolo, Renato Mariani-Costantini, Kerstin Leopold, Roland Schindl, Lavinia V. Lotti, Rocco Mangifesta, Enrico Sabbioni, Qiao Niu, Giovanni Bernardini, Mario Di Gioacchino

**Affiliations:** ^1^Immunotoxicology and Allergy Unit, Ce.S.I., “G. d'Annunzio” University Foundation, 66100 Chieti, Italy; ^2^Department of Medicine and Ageing Science, “G. d'Annunzio” University, 66100 Chieti, Italy; ^3^Unit of General Pathology, Ce.S.I., “G. d'Annunzio” University Foundation and Department of Medicine, Dentistry and Biotechnology, G. d'Annunzio University, 66100 Chieti, Italy; ^4^University of Ulm, Institute for Analytical and Bioanalytical Chemistry, 89081 Ulm, Germany; ^5^Laboratorio di Biopatologia Ultrastrutturale, Department of Experimental Medicine, University of Rome “La Sapienza”, 00161 Rome, Italy; ^6^Shanxi Medical University, Taiyuan, China; ^7^Department of Biotechnology and Molecular Sciences, University of Insubria, 21100 Varese, Italy; ^8^The Protein Factory, Interuniversity Centre Politecnico di Milano, ICRM CNR Milano and University of Insubria, 20131 Milan, Italy

## Abstract

There is concern about the possible toxicity of palladium nanoparticles (Pd-NP), as they are released in the environment through many applications. We previously studied the toxicity of Pd-NP at high concentrations; here we address the possible toxicity of Pd-NP at low, subtoxic doses. In particular, we have exposed normal human PBMC entering into the first in vitro mitotic division to Pd-NP and to Pd(IV) ions to evaluate ROS generation and cell cycle progression. We have measured a statistically significant increase of intracellular ROS in Pd(IV) exposed cells, but not in Pd-NP exposed cells. TEM revealed accumulation of lipid droplets and autophagic and mitophagic vacuoles, which appeared more conspicuous in cells exposed to Pd(IV) ions than to Pd-NP. Pd-NP were visible in the cytoplasm of Pd-NP exposed cells. Pd-NP addition was associated with a significant increase of cells within the G0/G1-phase and a significant reduction in GS- and G2/M-phases. Cells exposed to Pd(IV) ions showed a significant amplification of these cell cycle alterations. These results suggest that ions, per se or released by NPs, are the true inducers of Pd toxicity. It will be essential to verify whether the observed disturbance represents a temporary response or might result in permanent alterations.

## 1. Introduction

Palladium (Pd) is a component of the catalytic converters that promotes reduction and oxidation of pollutants in exhaust gases to less harmful ones [1]. Catalytic converters emit Pd mainly as particulate matter in the breathable size range (*d* < 2.5 *μ*m) [2, 3], containing also Pd-NP (*d* < 100 nm) [4]. Indeed, after the introduction of catalytic converters based on the Pt group elements, the level of Pd (also in the form of nanoparticles) rapidly increased in the areas bordering roads [5–10]. At the same time, in Europe, allergic sensitization to Pd increased [11–14]. A causative role for Pd(II) ions in the development of allergic symptoms has been deduced from studies on occupationally exposed people [14–16] and people wearing Pd-containing dental alloys [17] and is sustained by laboratory data [18]. Pd being chemically related to nickel, one of the most important sensitizing metals, phenomena of cross-reactivity might contribute to the increased frequency of immune reactions to Pd [19–21]. Few data are available to explain the role of Pd-NP for the allergic sensitization. Our recent data confirm the sensitizing power of Pd(II) and Pd(IV) ions [22, 23]. Moreover, we observed that Pd-NP can induce secretion of INF-*γ*, a Th1 cytokine involved in type IV immune reactions [24] suggesting a role for polluting Pd-NP in the raised frequency of allergic contact dermatitis to Pd in people living in urban settings [14].

On the other hand, no data are available for the possible involvement of Pd in cancer development, which is one of the major concerns about metal-based NPs. In the ionic form, Pd(II) does not induce significant genotoxicity [25], but no extrapolations are possible to Pd-NP. In fact, NPs have peculiar physical properties that may not be simply predicted by data from the corresponding ions. However, ions can be released from NPs and contribute to NP toxicity. Cobalt represents an example of these events. Recently, our group found that highly cytotoxic Co^2+^ ions are released from Co-NPs, but only nanoparticles are rapidly accumulated within the nucleus and interact with DNA [26], induce transformation [27], and affect molecular pathways implicated in carcinogenesis and inflammation [28]. All these findings were obtained at rather high exposure concentrations. In the present work, we have studied the effects of low, subtoxic Pd-NP doses. As a target, we used cells of the immune system, taking into account that palladium can act as hapten able to induce an immune reaction. In particular, to study the possible interference of Pd-NP in the cell cycle progression, we have exposed to Pd-NP normal human PBMCs entering the first in vitro mitotic division.

## 2. Materials and Methods

### 2.1. Palladium Model Nanoparticles and Palladium Ions

Zerovalent Pd-NP (2–8 nm diameter) were produced and characterized as described previously [29]. The Pd-NP stock solution did not contain Pd ions, even after long time from preparation, as assessed by low resolution inductively coupled plasma mass spectrometry. To exclude the presence of agglomerates, the Pd-NP stock solution was treated by ultrasonic bath for 10 min at 100 Hz (Elmasonic S, Elma, Singen, Germany) and then immediately diluted in complete culture medium at working concentrations and given to the cells straight away. Potassium hexachloropalladate (K_2_PdCl_6_, Sigma Aldrich, Milan, Italy) (Pd(IV) ion) was used at subtoxic concentration, for comparison.

### 2.2. Cells

Human peripheral blood mononuclear cells (PBMCs) from 3 healthy donors were isolated by density gradient centrifugation using a commercial separation medium consisting of a mixture of Ficoll 400 and sodium diatrizoate (Lymphoprep, StemCell Technologies, Voden Medical Instruments, Milan, Italy), according to standard manufacturer procedure. Cells were seeded at 500,000 cells mL^−1^ in RPMI-1640 medium supplemented with 10% fetal calf serum (complete culture medium) containing 5 *μ*g mL^−1^ phytohemagglutinin-L (PHA-L) (Sigma Aldrich, Milan, Italy), an initiator of mitosis of normal human leukocytes. To evaluate the effect of the mitogen on cell viability and number, the PHA-stimulated cells were examined every 24 h for 5 days by trypan blue exclusion using a Neubauer counting chamber as described [30]. After 48 hours of culture in the presence of PHA, Pd-NP (or Pd(IV) ions or an equal volume of the vehicle, as controls) were added to the cultures for further 48 hours and finally harvested appropriately for analysis. Experiments were done in triplicate.

### 2.3. Cytotoxicity Evaluation

Cytotoxicity was assessed by MTT (3-[4,5-dimethythiazol-2-yl]-2,5-diphenyl tetrazolium bromide) test according to the standard procedure. Briefly, PBMCs (40,000 per well in 200 *μ*L culture medium, four replicates) were seeded in a 96-well culture plate, incubated at 37°C, 5% CO_2_, and exposed to increasing concentrations of Pd-NP or Pd(IV) ion (0.1, 1, 5, 10, 20, 40, 80 *μ*g mL^−1^) or to an equal volume of vehicle (RPMI, 10% FCS) to assess 100% viability.

At the end of the exposure time, a washing step was performed to remove the incubation medium containing unreacted MTT and Pd-NP or Pd(IV) ion. Then, 20 *μ*L of the MTT solution (5 mg mL^−1^) was added to each well and incubated for an additional 3 hours. The colored formazan crystals produced from MTT reduction were dissolved in 200 *μ*L of dimethyl sulfoxide (DMSO). The optical density (O.D.) values of the solutions were measured at 540 nm as excitation wavelength using a spectrophotometer plate reader (Applied Biosystems, Life Technologies, Monza, Italy). Also, negative controls (i.e., complete culture medium alone or complete culture medium containing Pd-NP) have been tested and no interference with the colorimetric assay was observed, at all concentrations used. Cells without any treatment were assumed as positive control (100% viability).

The highest concentration that, after 48 h incubation, caused signal reduction (cell death) by less than 10% compared to the control was used for the experiments.

### 2.4. Detection of Intracellular Reactive Oxygen Species

The production of intracellular reactive oxygen species (ROS) was indirectly measured using dichlorodihydrofluorescein diacetate (DCFH-DA), a nonfluorescent and cell-permeant substance converted by endogenous esterases to dichlorofluorescein (DCFH), and nonfluorescent nonpermeant compound that cellular ROS oxidize proportionally to fluorescent dichlorofluorescein (DCF). Briefly, 200,000 PBMCs per well were seeded, in quadruplicate samples. After 48 hours, cells were washed with phosphate-buffered saline (PBS) and incubated with 10 *μ*M DCFH-DA, for 30 min, at 37°C. Finally, the fluorescence emitted by the oxidized form of DCFH-DA was measured using a CytoFluor fluorescence multiwell plate reader (Applied Biosystems, Life Technologies, Monza, Italy) with the excitation fluorescence wavelength at 485 nm and the emission one at 530 nm.

### 2.5. Transmission Electron Microscopy

PBMCs were fixed overnight at 4°C in 2% paraformaldehyde and 2% glutaraldehyde in PBS, postfixed in 1% osmium tetroxide in veronal acetate buffer (pH 7.4) for 2 h at room temperature, stained with 2% uranyl acetate, dehydrated in acetone, and embedded in Epon 812. Thin sections were examined under a Philips CM10 TEM after poststaining with uranyl acetate and lead hydroxide.

### 2.6. Cell Cycle Assay

Cells were fixed in 70% cold ethanol, stained with 50 *μ*g/mL propidium iodide in PBS buffer containing 200 *μ*g/mL RNase (DNase-free, Sigma Aldrich, Milan, Italy), and analyzed using a FACSCalibur flow cytometer equipped with the CellQuest software (Becton Dickinson). Debris were excluded from the analysis after gating them out by setting the forward scatter versus side scatter plot on the viable cells area. Cell doublets and aggregates were excluded by gating FL2-area versus FL2-width. The low flow rate mode (400–500 events sec^−1^) was used to record 20,000 nondebris events for each sample. PI fluorescence data were collected using the linear amplification mode. DNA content was assessed by placing the G1 peak at channel 400. Finally, data were analysed using ModFit LT software (Verity Software House, Toshan, ME, USA).

### 2.7. Statistical Analysis

Statistical analysis was performed using GraphPad Prism 5.0 software (La Jolla, CA, USA). MTT and ROS data were analyzed by Student's *t*-test; cell cycle data were analyzed by three- and four-way analysis of variance (ANOVA) with Tukey multiple comparisons correction, when appropriate. The comparison between the different groups in different concentrations was performed with the *t*-test. In all cases the threshold for significance was set at *P* < 0.05.

## 3. Results

**Figure 1:**
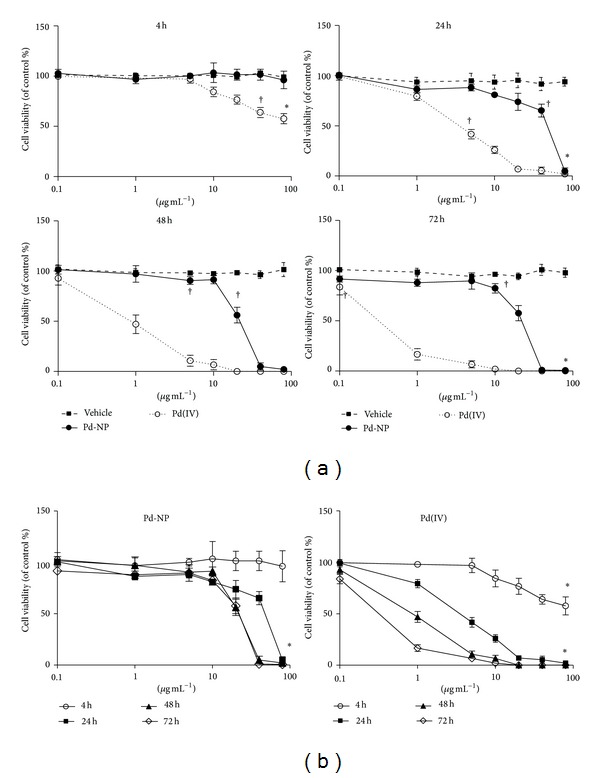
Evaluation of in vitro cytotoxicity by MTT assay. Different concentrations of Pd-NP (circles) (0.1, 1, 5, 10, 20, 40, and 80 *μ*g mL^−1^) or Pd(IV) ions (open circles) were added to PHA-stimulated PBMCs from healthy donors (*n* = 3) on the 3rd day of culture, in quadruplicate wells. As reference, parallel control wells were set by adding the vehicle alone (culture medium, 10% FCS) (squares). The extent of cell growth was measured after 4, 24, 48, and 72 hours of incubation. The vehicle did not significantly affect cell viability. (a) Data comparing the different compounds (vehicle, Pd-NP, and Pd(IV)) are plotted in logarithmic scale and shown as mean ± S.D., as a function of concentration. (b) Data comparing the incubation time for Pd(IV) and Pd-NP are plotted in logarithmic scale and shown as mean ± S.D., as a function of concentration. The differences in cell viability related to Pd(IV) versus vehicle reached statistical significance (*P* < 0.05) starting from the following concentration/time exposure combinations: 40 *μ*g mL^−1^ after 4 hours, 5 *μ*g mL^−1^ after 24 h, 1 *μ*g mL^−1^ after 48 h, and 0.1 *μ*g mL^−1^ after 72 h. Pd-NP-induced cytotoxicity was statistically significant (*P* < 0.05) versus vehicle for the following conditions: 80 *μ*g mL^−1^ after 24 h, 20 *μ*g mL^−1^ after 48 h, and 10 *μ*g mL^−1^ after 72 h. *P < 0.001 three-way ANOVA with Tukey correction, ^†^ *P* < 0.05  *t*-tests Pd-NP and Pd(IV) vs not exposed.

PBMCs, which are recognized as quiescent cells (G0 state), were induced to enter the cell cycle by in vitro stimulation with PHA, a well-known mitosis inducer. After a latency period of 48 hours, the number of cells started to increase and doubled within further 72 hours. Pd-NP were added 48 hours after PHA, at the beginning of the growth phase. After 4 hours of exposure to Pd-NP, no evident changes in cell viability were observed at any tested dose. Prolongation of the exposure time to 24 hours showed a concentration-dependent decrease of viability up to 40 *μ*g mL^−1^ and death at 80 *μ*g mL^−1^. At 48 hours, also 40 *μ*g mL^−1^ treated cells were incapable of metabolic conversion of MTT, and the same result was obtained after 72 hours of exposure. The viability data for Pd-NP are shown in Figure 1. Pd(IV) ions were more toxic, causing almost complete loss of cell viability earlier, at 48 hours and at lower dose (5 *μ*g mL^−1^). Therefore, the following experiments were performed using the nontoxic doses of 10 *μ*g mL^−1^ Pd-NP and 0.1 *μ*g mL^−1^ Pd(IV) ions, for 48 hours.

**Figure 2:**
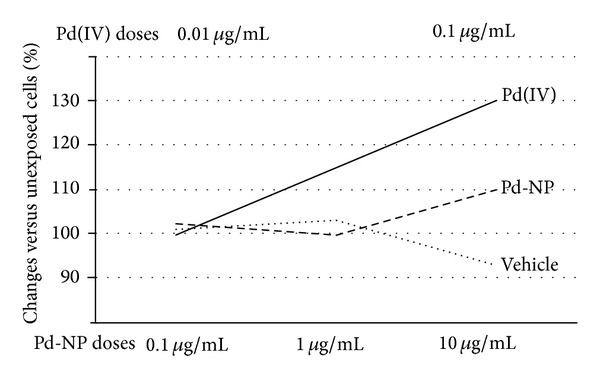
ROS production. Production of ROS by cells exposed to vehicle was similar to that of unexposed cells. Nontoxic concentration of Pd-NP induced a moderate, but not significant, change in ROS production in respect to unexposed and vehicle exposed cells, whereas cells stimulated by Pd(IV) ions showed a significant increase in ROS production (by 30% in respect to unexposed cells. *P* < 0.05).

**Figure 3:**
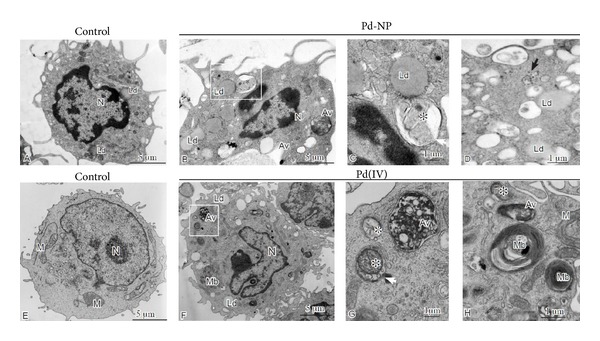
Ultrastructural alterations observed in peripheral blood mononuclear cells exposed to Pd-NP and ions. The upper panels show alterations observed in cells exposed to 10 *μ*g mL^−1^ Pd-NP (B-D) relative to unexposed control (A); the lower panels (F-H) exemplify, relative to control (E), the alterations seen in PBMCs exposed to 0.1 *μ*g mL^−1^ Pd (IV) ions. PBMCs exposed to cobalt NPs and ions show numerous autophagic vacuoles (Av) at different stages of maturation, often enclosing damaged mitochondria (indicated by asterisks in C and G and white arrow in G). Accumulation of lipid droplets (Ld) and multilamellar bodies (Mb), membrane-bound structures composed of concentric membrane whorls, is also readily evident. Agglomerates of electron-dense nanosized particles, consistent with internalized Pd-NP, are present within or near cytoplasmic vacuoles in PBMCs (indicated by black arrows in D). Compared to the untreated controls, the PBMCs exposed to Pd-NP and ions show increased membrane ruffling and more indented nuclei (B and F).

A moderate, but not statistically significant increase of intracellular ROS was measured both in controls and in Pd-NP exposed cells, whereas ROS significantly (*P* < 0.05) increased after Pd(IV) exposure by more than 30% of the control (Figure 2). Electron microscopy revealed that, as compared to controls, PBMCs exposed to Pd-NP and to Pd ions shared marked subcellular alterations (Figure 3). Most notably, these included the presence of numerous autophagosomal vacuoles containing damaged mitochondria and/or undigested cytoplasmic material, as well as the accumulation of multilamellar bodies and lipid droplets. Such alterations were more marked in PBMCs exposed to Pd ions, being already well evident at our nontoxic concentration. With both treatments, mitochondria showed evidence of damage, as indicated by condensation and loss or swelling of cristae. Furthermore, in both cases, there was evidence of increased plasma membrane ruffling and nuclei were more indented and/or convoluted compared to controls. In PBMCs exposed to Pd-NP, electron-dense agglomerates of nanosized particulate material within or adjacent to cytoplasmic vesicles were consistent with the internalization of Pd-NP.

**Figure 4:**
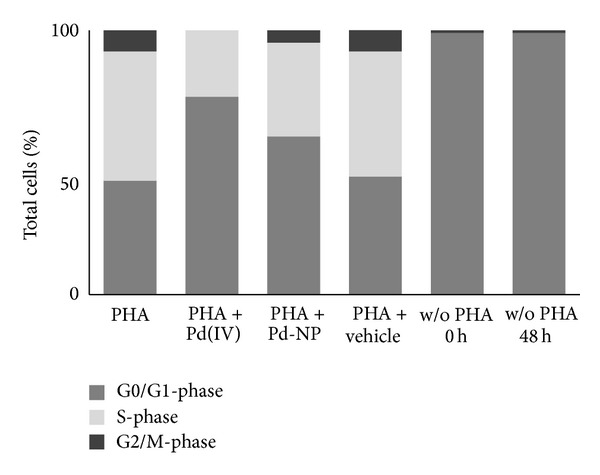
Cell cycle analysis of human PBMCs exposed to Pd-NP. PBMCs (*n* = 3) were cultivated in the presence or absence of PHA for 48 hours and after being exposed or not to Pd-NP (10 *μ*g mL^−1^) or Pd(IV) ion (50 ng mL^−1^) for 48 hours. For comparison, an equal volume of vehicle was used in parallel cultures. The proportion of cells in the different phases was quantitated using ModFit software and represented with partitioned bars as the average of three experiments. G0/G1-phase: dark grey; S-phase: light grey; G2/M-phase: black.

The examination of cell cycle (Figure 4) confirmed the quiescent/resting state of all unstimulated PBMCs either freshly isolated (w/o PHA, 0 hours) or cultured for the entire experimental period (w/o PHA, 96 hours), as they were found in the G0 state. The addition of the polyclonal activator PHA stimulated cell division, as shown by their distribution throughout all phases of the cell cycle (G0/G1: 43.1 ± 1.9%; S: 48.8 ± 2.3%; G2/M: 8.1 ± 1.2%). The exposure of PHA-activated PBMCs to 10 *μ*g mL^−1^ Pd-NP for the time allowing the completion of at least one cell division (48 h) was associated with a significant reduction of cells synthesizing DNA (S-phase) (35.4 ± 1.5% versus 48.8 ± 2.3%, *P* < 0.05), a significant increase of cells within the G0/G1-phase (59.8 ± 1.8% versus 43.1 ± 1.9%, *P* < 0.05), and a significant reduction of cells in G2/M-phase (4.8 ± 0.1% versus 8.1 ± 1.2%, *P* < 0.05), compared with unexposed control cells. A different cell cycle outline was observed for Pd(IV) ion exposed cells, with significantly higher G0/G1 accumulation compared to Pd-NP exposed cells (74.9 ± 2.2% versus 59.8 ± 1.8%, *P* < 0.05), lower percent of cells in S-phase (25.1 ± 1.2% versus 35.4 ± 1.5%, *P* < 0.05), and no cells engaged in the cell division (G2/M). The vehicles alone did not cause significant changes of the cell cycle distribution. Also, PBMCs were evaluated for cell cycle distribution after exposure to Pd-NP and Pd(IV), in absence of PHA stimulation; in no case were these compounds found to induce cell cycle initiation and all cells remained in the G0 phase (data not shown).

Sub-G0/G1 cells, indicative of cell death, were largely absent from the analyzed populations, for all tested samples. All cell cycle data are summarized in (Table 1).

## 4. Discussion

In the present work, we have found that Pd-NP exposure is associated with an increased percentage of mitogen-activated PBMCs with a diploid DNA content, indicative of maintenance of G0 state or prolongation/arrest in G1-phase. Studying the influence of potential toxicants on the G1-phase of the cell cycle is of central importance; in fact, most, if not all, human cancer types show a deregulated control of G1 progression, a period in which cells decide whether to start proliferation or stay quiescent [31, 32]. Important processes, such as increase in cell size and centrosome duplication, are initiated in G1 and are thus controlled. Control is conducted by checkpoint complexes that stall the cell cycle until the appropriate repair is completed. If such repair is not achieved, the cell may pause or undergo senescence or apoptosis. Alteration of repair (controlled by caretakers) and checkpoint complexes (controlled by gatekeepers genes) is central in oncogenesis [33].

In our experiments, PBMCs, which are mostly quiescent cells in the G0 state and prompted to enter the cell cycle by in vitro stimulation with PHA, were exposed to low noncytotoxic dose-time combinations of Pd-NP and Pd ions. Under these experimental conditions, Pd-NP gain entry to the cytosol, where they appear in clusters lacking a membranous envelope, suggesting the possibility to exert a catalysing redox activity [34] on cellular elements.

At the ultrastructural level the alterations observed in the PBMCs exposed to Pd-NP largely corresponded to those observed in the PBMCs exposed to Pd ions. Most notably, in both cases the cells showed evidence of mitochondrial damage and of mitophagy, that is, autophagic elimination of damaged mitochondria. Mitophagy is a key protective mechanism against mitochondrial damage and the consequent ROS-induced cellular alterations [35]. The accumulation of abundant multilamellar bodies is also in agreement with the macroautophagic response, as these structures originate via autophagy and reflect the accumulation of membrane lamellar material selectively resistant to lysosomal degradation within autophagolysosomes. Lipid droplets, specialized organelles for the deposition and storage of neutral lipids, are associated with common pathologies linked to lipid accumulation and mitochondrial damage. Their accumulation in PBMCs exposed to Pd is fully consistent with the loss of mitochondrial fatty acid beta oxidation due to mitochondrial damage, that, in turn, results in lipid accumulation and loss of ATP production.

In conclusion, the morphological evidence strongly suggests that Pd ions, per se or dissolved from NPs, accumulate in the mitochondria, as we recently demonstrated in the case of cobalt [26]. Here, acting as catalyst, ionic Pd may interfere with the oxidative phosphorylation (OXPHOS). The mitochondria, whose respiratory activity is damaged by the influx of Pd ions, will be poor ATP producers and will release excessive amounts of ROS. The ROS-induced cellular stress is predicted to arrest the cell cycle, acting via oxidative stress-sensitive cell cycle regulator genes such as P53 and CDKN2a, which we have recently found to be implicated in the response to Co particles in Balb/3T3 cells [28]. In turn, ROS and ATP depletion activate cell death pathways, which induce the release of proapoptotic proteins from mitochondria. Macroautophagy may mitigate these effects by the selective sequestration and subsequent degradation of the dysfunctional mitochondria and other ROS-damaged subcellular structures before the activation of prodeath pathways [30, 35, 36]. Thus, macroautophagy, and, in particular, mitophagy, most likely represents a key prosurvival pathway in cells exposed to palladium.

Autophagy has been demonstrated to be marker of heavy metals toxicity for human hematopoietic progenitor cells [30], representing a protecting and recycling catabolic pathway removing damaged organelles and molecules [37]. The cellular outcome of inducing autophagy in response to stress is complex and depends on the cellular context, type, and magnitude of stress. In mild stress conditions, autophagy may enhance cell survival by allowing the cell to engage DNA repair mechanisms and checkpoint activation [38]. Nontoxic dose of Pd ions induced a more marked production of ROS relative to Pd-NP, contrary to almost all other metal-based NPs [39]. However, local ROS generation at sites of Pd-NP accumulation, not measurable by the overall method used, might be possible at the nontoxic doses applied in our experiments. In fact, local concentration is important for the cellular function of ROS [40]. On the other hand, changes in ROS generation may modulate autophagic processes, as ROS can modify activating molecules to stimulate or inhibit autophagy and engages in cross-talk with autophagy in both cell signaling and protein damage [41].

Mechanisms of cell damage by Pd-NP can be also explained through cell cycle disturbance by the withdraw/inactivation of nutrients and/or PHA or by modulation/inhibition of downstream signaling due to their catalytic activity, as described for polystyrene-coated Pd nanoparticles [34]. Therefore, Pd-NP might produce an effect similar to nutrient depletion in inducing autophagy in the G1 phase [42]. It is possible that Pd-NP interference with mitogen/growth factors would prolong the G1-phase checkpoint called restriction point that prevents cells from entering the cycle until they have accumulated a certain threshold of mitogen-induced events [43].

## 5. Conclusions

The described findings suggest a potentially harmful role for low dose Pd ions and Pd-NP in hematopoietic cells leaving quiescence. However, it will be essential to verify whether the observed disturbance of the transition from quiescence to cell division represents a temporary response or might lead to permanent alterations of the cell functions. To this aim, long-term exposure to low dose of Pd (ions and NPs) and single cell-based technologies are envisaged.

## Figures and Tables

**Table 1:** Cell cycle distribution data of quiescent and PHA-activated PBMCs exposed to Pd-NP or Pd(IV) ion.

Phase	PHA	PHA + Pd(IV) ion	PHA + Pd-NP	PHA + vehicle	w/o PHA 0 h	w/o PHA 48 + 48 h
G0/G1	43.1 ± 1.9	74.9 ± 2.2∗	59.8 ± 1.8∗	44.4 ± 2.1	98.9 ± 0.2	99.0 ± 0.1
S	48.8 ± 2.3	25.1 ± 1.2∗	35.4 ± 1.5∗	47.3 ± 1.7	0	0
G2/M	8.1 ± 1.2	0	4.8 ± 0.1	8.3 ± 1.4	0.9 ± 0.2	1.0 ± 0.1

Data from three experiments are shown as mean ± S.D. ∗*P* < 0.05 Pd-NP and Pd(IV) vs not exposed.
